# Social bonding between humans, animals, and robots: Dogs outperform AIBOs, their robotic replicas, as social companions

**DOI:** 10.1371/journal.pone.0324312

**Published:** 2025-06-03

**Authors:** Stella Klumpe, Kelsey C. Mitchell, Emma Cox, Jeffrey S. Katz, Lucia Lazarowski, Gopikrishna Deshpande, Jonathan Gratch, Ewart J. de Visser, Hasan Ayaz, Xingnan Li, Adrian A. Franke, Frank Krueger

**Affiliations:** 1 Institute of Clinical Psychology and Psychotherapy, University of Technology Dresden, Germany; 2 School of Systems Biology, George Mason University, Virginia, United States of America; 3 Department of Psychological Sciences, Auburn University, Alabama, United States of America; 4 Department of Electrical & Computer Engineering, Auburn University Neuroimaging Center, Auburn University, Alabama, United States of America; 5 Canine Performance Sciences Program, College of Veterinary Medicine, Auburn University, Alabama, United States of America; 6 Department of Anatomy, Physiology, and Pharmacology, College of Veterinary Medicine, Auburn University, Alabama, United States of America; 7 USC Institute for Creative Technologies, California, United States of America; 8 Warfighter Effectiveness Research Center, United States of America Air Force Academy, Colorado, United States of America; 9 School of Biomedical Engineering, Science and Health Systems, Drexel University, Pennsylvania, United States of America; 10 Department of Psychological and Brain Sciences, College of Arts and Sciences, Drexel University, Pennsylvania, United States of America; 11 A.J. Drexel Autism Institute, Pennsylvania, United States of America; 12 University of Hawaii Cancer Center, Hawaii, United States of America; Universitat de Girona, SPAIN

## Abstract

In the evolving landscape of technology, robots have emerged as social companions, prompting an investigation into social bonding between humans and robots. While human-animal interactions are well-studied, human-robot interactions (HRI) remain comparatively underexplored. Ethorobotics, a field of social robotic engineering based on ecology and ethology, suggests designing companion robots modeled on animal companions, which are simpler to emulate than humans. However, it is unclear whether these robots can match the social companionship provided by their original models. This study examined social bonding between humans and AIBOs, dog-inspired companion robots, compared to real dogs. Nineteen female participants engaged in 12 affiliative interactions with dogs and AIBOs across two counter-balanced, one-month bonding phases. Social bonding was assessed through urinary oxytocin (OXT) level change over an interaction, self-reported attachment using an adapted version of the Lexington Attachment to Pets Scale, and social companionship evaluations administering the Robot-Dog Questionnaire. To examine OXT level changes and self-reported attachment by comparing the two social companions, we conducted mixed-effects model analyses and planned follow-up comparisons. Frequency comparison, binary logistic regression, and thematic analysis were performed to analyze social companionship evaluations. Results revealed significant differences between dogs and AIBOs in fostering social bonds. OXT level change increased during interactions with dogs but decreased with AIBOs. Participants reported stronger attachment to dogs and rated them as better social companions. These findings highlight the current limitations of AIBOs in fostering social bonding immediately compared to dogs. Our study contributes to the growing HRI research by demonstrating an existing gap between AIBOs and dogs as social companions. It highlights the need for further investigation to understand the complexities of social bonding with companion robots, which is essential to implement successful applications for social robots in diverse domains such as the elderly and health care, education, and entertainment.

## 1 Introduction

### 1.1 Social bonding and oxytocin in human-robot interactions

In recent decades, technology has become increasingly integrated into our daily lives, manifesting in various forms and applications. However, the emergence of social robots as everyday companions for humans signals a new era in human-robot interactions (HRI). It raises the question of whether social bonds, characterized as lasting, positive, and affiliative social relationships, can arise from this new form of liaison [1]. A sophisticated understanding of technical and human social capabilities is needed to establish these interactions and explore the potential of forming interspecific social bonds. This requires drawing from a framework that encompasses biological, psychological, and technological insights into underlying mechanisms [2].

While previous research has extensively explored interactions among humans and between humans and animals, particularly the profound relationship between humans and dogs, the field of HRI remains comparatively underexplored [3,4]. However, understanding the intricate dynamics of human-robot relationships is essential for unlocking the full potential of social robots as companions, caregivers, and collaborators across diverse domains such as the elderly and health care, education, and entertainment [5]. Identifying the fundamental factors that underpin social bonding and attachment can offer valuable insights into the mechanisms driving the establishment and nourishment of successful HRI. This understanding can pave the way for developing robots capable of forging deep and meaningful relationships with humans [6–11].

One possible starting point to explore social bonding in HRI is a neurobiological approach focusing on the neuropeptide oxytocin (OXT) [12]. Renowned for fostering social bonding among humans and other mammalian species, OXT profoundly influences various social behaviors and affiliative processes [13–16]. OXT plays a pivotal role in promoting social bonds among individuals of the same species, among others, enhancing the perception of bonding behaviors and recognition of facial emotions, increasing pro-sociality, and mitigating stress responses in the presence of positively perceived social cues [17–21]. Its impact also extends to interspecies relationships by facilitating social bonds between humans and animals such as dogs [22–25]. Taken together, OXT can reflect the establishment and consolidation of social bonds between con- and heterospecifics on a neurobiological level and, therefore, can serve as an indicator of successful bonding in HRI.

One exemplary study investigating the formation of social bonds between humans and companion robots using OXT as a neurobiological bonding indicator found that individuals living with a companion robot for more than two months exhibited more affiliative behaviors and higher steady-state levels of urinary OXT in comparison to individuals who did not live with a companion robot [26]. However, while this study showed that the initiation of social bonding was successful compared to living with no type of social companion, the question remains whether this positive change is subject to a genuine bonding experience or to a mere-exposure effect, i.e., developing liking with increasing familiarity. This effect was already reported in prior research, which showed improved owner ratings of their social robot over time [27]. An active control condition is needed to better understand the nature of the observed changes in social bonding parameters, allowing an immediate comparison between a social robot and a conventional social companion such as a dog.

### 1.2 Social bonding and companionship in ethorobotics

Humans and dogs share a uniquely long period of coevolution spanning over 10,000 years [28]. During this domestication process, dogs have developed unique socio-cognitive abilities to communicate with humans, which have not been observed in great apes or human-reared wolves [29]. This exceptional set of evolved social skills includes the ability to interpret human cues, exhibit emotional expressions, and engage in reciprocal interactions, which, together with the individual development of human-like personality traits, dogs can form deep emotional attachments with humans [30–35]. By leveraging these qualities, dogs can serve as a valuable model for informing the design of socially intelligent robots capable of engaging and establishing meaningful connections with humans [36–38]. This aligns with the scientific approach of ethorobotics, an interdisciplinary approach to social robotic engineering that focuses on ecology and ethology and strives to maximize the performance of social robots specific to their purpose instead of merely mimicking humans [5]. While previous HRI research predominantly attempted to base social robots on human-human interactions, the field of ethorobotics proposes human-animal interactions as a more suitable source for building behavior and communication models of social robots due to sufficient but less complex cognitive and physical capabilities of animals compared to humans [39,40]. Taken together, dogs are well-established social companions for humans and, thus, valuable informants on social behaviors and suitable comparable subjects in research aiming to disentangle social bonding and familiarity-based liking in HRI and to survey the progress of robots in social companionship.

One example of a dog-inspired social robot is AIBO, an acronym for Artificial Intelligence roBOt, meaning “friend”, “pal”, or “companion” in Japanese. AIBO is an AI-powered robotic dog imitation designed as a social companion by Sony Corporation (https://us.aibo.com/). AIBO facilitates social interactions via advanced sensory systems and AI-powered algorithms registering various environmental cues (e.g., human voice commands) and modeling social behaviors (e.g., tail wagging) that enable reciprocal communication and engagement with humans [41]. As AI technology advances, social robots like AIBO have the potential to reshape our understanding of social interactions and redefine the boundaries of companionship in the modern technological landscape. This potential can be realized if biological, psychological, and technological insights are integrated to inform and guide their design process. Therefore, research exploring these dimensions of HRIs can contribute to developing social robots that better comprehend and respond to human emotions, needs, and preferences, fostering genuine social bonding and effective collaborations between humans and robots. The design of AIBO emulates the social characteristics and behaviors of dogs while maintaining a robotic appearance. Following the approach of ethorobotics, AIBO thus enables a direct comparison of its ability to facilitate social bonds with humans to that of its animal model, providing valuable insights into HRI based on human-animal interactions.

Previous literature contrasting companion robots and dogs revealed that humans tend to have a negative attitude and a lack of interest toward companion robots, with the majority of people stating the belief that robots cannot be as good or loved as much as companion dogs [42]. Furthermore, it was shown that emotions, personality, and the display of attachment are dogs’ most liked features compared to companion robots, constituting highly complex psychological constructs that are difficult to convincingly model in robots [43]. However, AIBO was designed to better meet human expectations and demands toward social companions at the level of dogs, and thus, it might undermine previous research results as being outdated. Even an earlier model of AIBO was found to be socially engaging due to its successful illusion of animacy. However, it was still perceived as a technological artifact, enabling a one-sided relationship that could be paused at any time [44]. Although the current AIBO model holds more sophisticated technology and software and is based on more advanced research, it still might not be able to elicit social bonding to the same extent as companion dogs, suggesting a comparatively poorer bonding experience. This hypothesis aligns with previously discussed literature stating that positive results on social bonding with companion robots might substantially be subject to a mere exposure effect and that social bonding is commonly believed to work better with dogs than robots.

However, the quality and success of interactions with social robots are not solely determined by their abilities and features. On the human side, Openness to experience, a personality trait characterized as a need for variety, novelty, and change, has been found to influence the success of HRIs [45] significantly: individuals high in Openness tend to report a more positive experience with and greater acceptance of social robots [46–47]. Thus, more open individuals might tend to bond better with social robots, especially if these individuals are familiar with dogs but not with robots as social companions, which provides more novelties to explore. On the other hand, dogs might be able to better sustain the interest of more open individuals by displaying challenging behaviors and demanding more responsibilities compared to social robots designed for effortless social companionship [44]. Therefore, further insights into the role of Openness to experience in the dynamics of social bonding can contribute to a better understanding of robotic and animated social companions and their differences, potentially offering valuable guidance for improving the design of companion robots.

### 1.3 Study objectives and hypotheses

Our study aimed to contribute to the growing research on social bonding between humans and companion robots by investigating the development of social bonds with AIBO as an advanced dog-inspired companion robot compared to dogs as well-established animated social companions. We aimed to gather insights into the underlying process that led to elevated social bonding indicators in previous HRI studies, differentiating the formation of a genuine social bond from a mere-exposure effect and simultaneously testing the progress of robotic social companionship in contrast to a popular animated companion. Furthermore, we explored the influence of Openness to experience on interspecific social bonding and, thereby, discern possible resulting differences between robotic and animated social companions. Given that the development, consolidation, and maintenance of social bonds are driven by friendly and peaceful encounters, referred to as affiliative interactions [48], we compared the onset and short-term development of social bonding between human-AIBO and human-dog dyads over a series of affiliative interactions. We employed a cross-over design, consisting of two counter-balanced one-month bonding phases, each encompassing twelve affiliative interactions. The participants either started with a dog or an AIBO as bonding partners and switched at the End of the first bonding phase. We collected data on neurobiological and psychological markers of social bonding, such as urinary OXT, self-reported attachment, and evaluation of social companionship for each bonding partner. In addition, behavioral observations and fMRI scans were conducted, which will be the subject of a separate study report and are not further discussed here.

Based on the previous research findings, our overall hypothesis was that both bonding partners would initiate social bonding but that dogs would enable a more profound connection than their robotic counterparts. We expected to see this difference on three distinct levels indexing social bonding: neurobiological, emotional, and evaluative. Therefore, we predicted that urinary OXT levels are higher during the bonding phase with dogs (hypothesis I, H-I), self-reported attachment is greater to dogs (H-II), and dogs receive more positive evaluations of social companionship (H-III). Additionally, we explored the influence of Openness to experience social bonding on all three levels. Overall, our findings of this study provide preliminary support for our overall hypothesis, suggesting that social bonding works better with dogs than AIBOs.

## 2 Materials and methods

### 2.1 Subjects

Nineteen female students, ranging in age from 18 to 29 years (*M* = 20.33, *SD* = 2.5), were recruited through an e-mail distribution list for students by the Department of Psychological Sciences at Auburn University, AL, US. The recruitment period lasted from 15/04/2021 to 15/09/2021. Participants were randomly assigned to begin the first bonding phase with a companion pet (dog) or a companion robot (AIBO), completing the second bonding phase with the alternate companion. While participants were familiar with the general research topic of social bonding, they were blinded to the specific hypotheses being tested. The study was approved by the Auburn University Institutional Review Board (#21–059 MR 2102 Neural Correlates of Attachments in Human-dog versus Human-robot Interactions) and the Institutional Animal Care and Use Committee (#2021–3864 Neural Correlates of Attachments in Human-dog versus Human-robot Interactions). Written informed consent for study participation was obtained from all participants, who received academic credits in exchange for study participation.

### 2.2 Material and instruments

#### 2.2.1 Bonding partners.

Two types of bonding partners were employed to compare the social bonding of humans with a companion animal (represented by a dog) and with a companion robot (represented by an AIBO).

Dog. Subjects were six male and five female Labrador retrievers for this experiment from Auburn University's Canine Performance Sciences (AUCPS) program, purpose-bred and trained for odor detection work. Ages ranged from two to eight years (*M* = 5.5 years, *SD* = 1.44). Dogs were housed individually in kennels with indoor/outdoor runs at Auburn University’s College of Veterinary Medicine (AUCVM), which is an Association for Assessment and Accreditation of Laboratory Animal Care International accredited facility.

**Fig 1:**
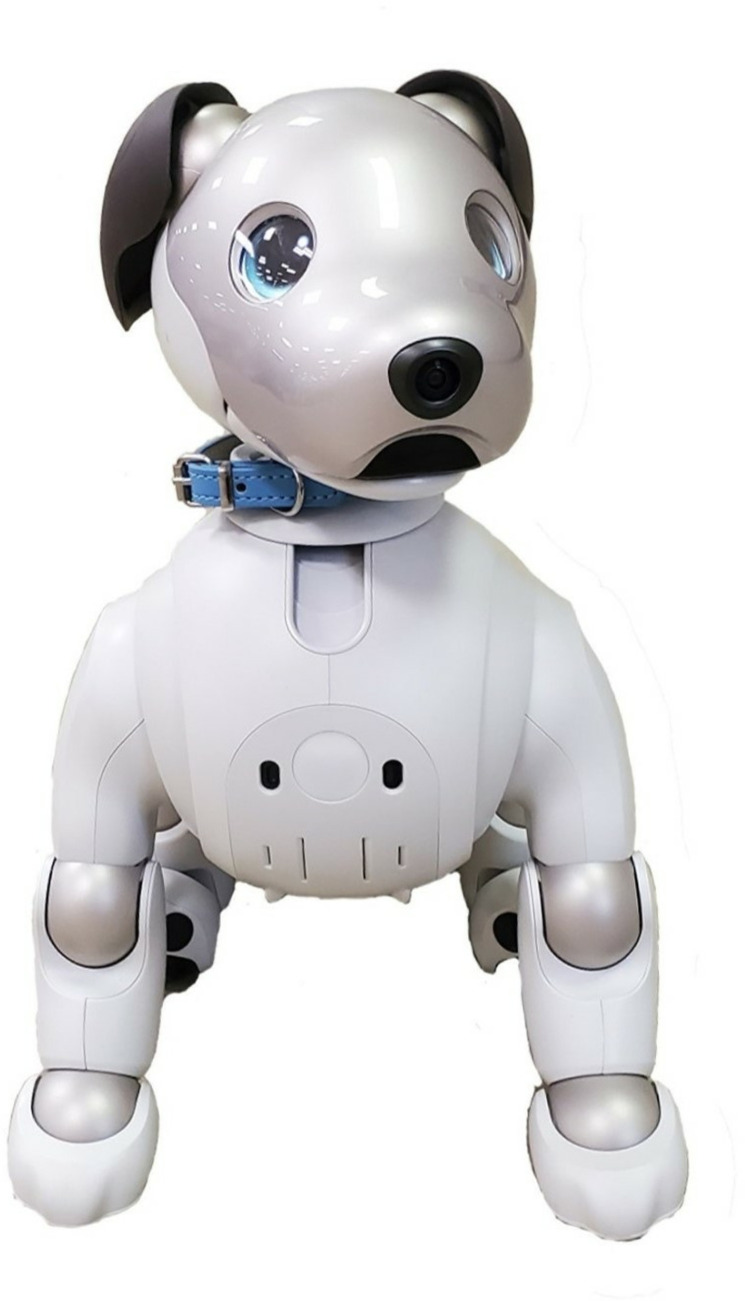
Exemplary Appearance of an AIBO Robot. This image illustrates an AIBO robot wearing a blue collar that served as a robotic social companion in this study.

AIBO. The latest AIBO model, ERS-1000, was released in 2018 (https://us.aibo.com/). It possesses a multimodal sensory system including, among other things, four microphones, two cameras on the nose and back enabling data input for voice, facial, and object recognition, as well as pressure sensors on the paws, and touch sensors on the head, back, and chin allowing behavioral responsivity to touch stimulation. AIBO’s repertoire of movements includes walking, sitting, lying down, shaking hands, and tricks such as dancing, and the built-in speaker allows it to play various sounds increasing communicational variability. The appearance of AIBO consists of dog-resembling (e.g., choice of body parts, eye design) and dog-distinct properties (e.g., design simplicity, surface colors, materials) (Fig 1). AIBO employs reinforcement learning for behavioral and personality development depending on and adapting to its environment. Its developmental state can be reset anytime to restart AIBO’s learning process from the beginning. AIBO’s communication capabilities encompass registration of and response to visual, voice, and touch cues, enabling it to differentiate between human individuals, learn and adhere to commands, and engage in interactive games such as fetch with dedicated toys (ball, bone, dice) and hide-and-seek. With the app *My Aibo*, users can additionally assign names, genders, and eye colors to their AIBO and feed it virtual food and water. Five AIBOs were employed in the experiment, while one additional AIBO was used for demonstration.

#### 2.2.2 Self-report questionnaires.

Self-report questionnaires were administered using Qualtrics (https://www.qualtrics.com) to collect relevant medical information, evaluate participants’ personality traits, assess participants’ subjective level of attachment, and collect social companionship evaluations of each bonding partner.

Medical Information. For the collection of medical data, a brief medical background questionnaire was administered, obtaining information on intake and type of medication, supplements, or birth control, the beginning of the last menstrual cycle, and the presence of medical diagnoses.

Personality Traits. The 50-item International Personality Item Pool (IPIP) representation of Goldberg’s markers for the Big-Five factor structure, measuring extraversion, agreeableness, conscientiousness, neuroticism, and openness, was administered to evaluate personality traits [49]. Participants indicated the level of accuracy for each first-person singular statement on a 5-point Likert scale ranging from “very inaccurate” (1) to “very accurate” (5). After recording reversed items, numerical values are summed up to five scale scores, and each is divided by the respective count of scale items, representing one trait.

Self-Reported Attachment. For the assessment of self-reported attachment, the Lexington Attachment to Pets Scale (LAPS) was adapted and administered [50]. The original LAPS consists of 23 items and measures the emotional attachment of individuals to their pets with three subscales: general attachment, people substituting, and animal rights/welfare. All items were rephrased for this study, and five items were entirely replaced. Participants indicated their level of agreement with each first-person singular statement on a 4-point Likert scale ranging from “strongly disagree” (0) to “strongly agree” (3). After re-coding reversed items, numerical values are summed up to a total emotional attachment score.

Social Companionship Evaluation. For the collection of social companionship evaluations, the Robot-Dog Questionnaire (RDQ) was administered [42]. The RDQ captures the individual participants’ opinions of dogs and robots by comparing their features and qualities. It consists of 28 items with 16 forced-choice and 12 open-ended response formats.

### 2.3 Setup and procedure

#### 2.3.1 Experimental setting.

**Fig 2:**
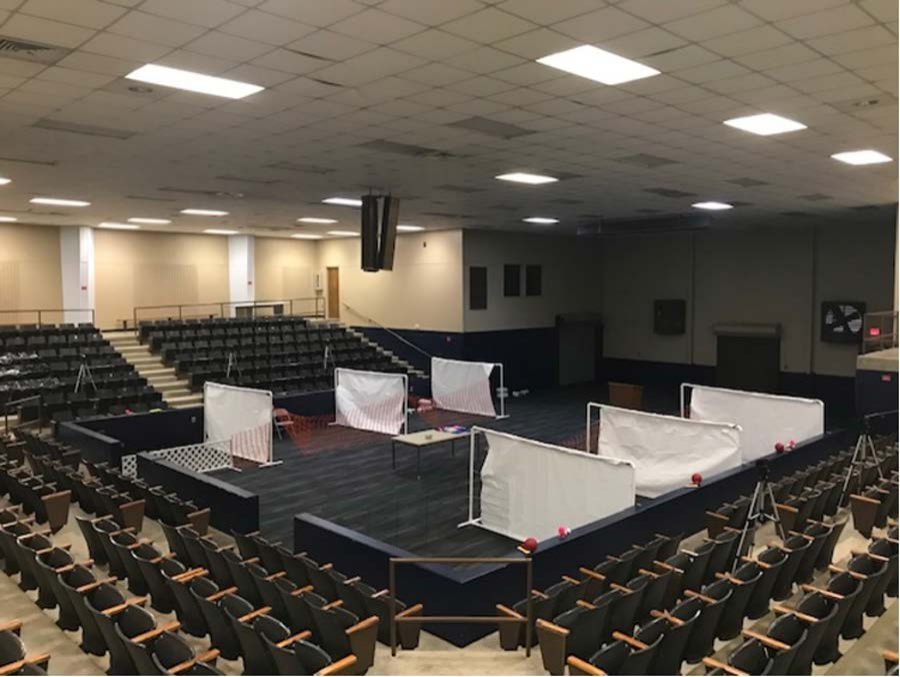
Experimental Environment. The experimental environment encompasses six individual stalls separated by constructed walls, allowing six participants to complete a session simultaneously.

The experimental environment provided room for six participants to simultaneously complete an interaction session in individual stalls separated by constructed walls (Fig 2). Three toys (ball, bone, dice), that AIBO can interact with, were placed in each stall before each interaction session. The dogs were provided with toys selected by the AUCPS staff. Video cameras were set up at each of the five stalls to capture the interaction for behavioral analysis and generation of fMRI task stimuli, which will be discussed further in separate articles. For each participant, the partnered AIBO was reset to its blank default learning state before the first encounter between participant and AIBO.

#### 2.3.2 Experimental procedure.

**Fig 3:**
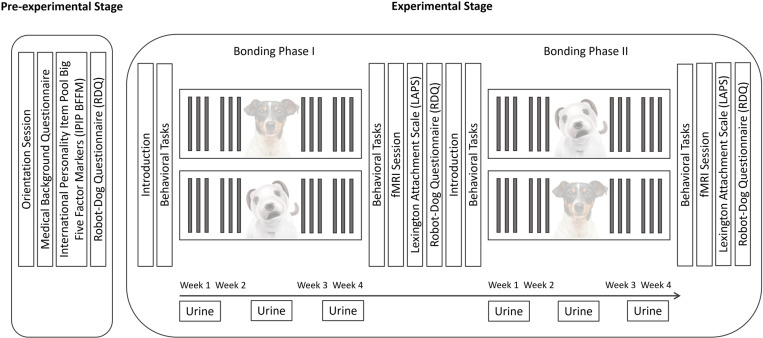
Study Procedure Divided in Pre-Experimental and Experimental Stage. In the pre-experimental stage, questionnaire data on participants’ medical background, personality, and opinion towards dogs and robots were collected, and an orientation session introducing AIBO and the study was conducted. The experimental phase consisted of two counter-balanced bonding phases, one with a dog and one with an AIBO, which each lasted for four weeks and encompassed twelve interaction sessions. Each bonding phase started with the introduction of the bonding partner and the conduct of behavioral tasks and ended with the completion of a questionnaire capturing attachment to the respective bonding partner, a repetition of the questionnaire collecting opinions towards dogs and robots and behavioral tasks, and an fMRI scanning session. During each bonding phase, urine samples were collected twice during the first, sixth, and twelfth interaction sessions, later referred to as time stages “Begin”, “Middle”, and “End”.

The study consisted of two stages, the pre-experimental and experimental stage (Fig 3). While the former served as study preparation, the latter contained a series of affiliative interactions with each bonding partner, behavioral tasks and two fMRI sessions.

Pre-Experimental Stage. Before the experimental stage started, participants were invited to an orientation session providing background information on the study and a short video showcasing and familiarizing participants with the capabilities of an AIBO. Subsequently, participants were allowed to choose the gender, name, eye color, and collar of their assigned AIBO. Before the first interaction session started, each participant received an e-mail with an invitation link to complete the questionnaires (medical background, IPIP, RDQ) online.

Experimental Stage. The experimental stage consisted of two bonding phases followed by an fMRI scanning session. Additionally, behavioral assessments of the attachment style of the respective bonding partner were conducted before and after each bonding phase.

Each bonding phase lasted one month, with three weekly interaction sessions consisting of petting, playing, and exercising familiar commands. Participants received a list of potential commands and games for interacting with AIBO, allowing interaction experiences comparable to those of dogs. During each bonding phase, a dog was only partnered with one participant, whereas each AIBO consecutively interacted with multiple participants due to its learning state reset function. Each participant was paired with a real dog and an AIBO.

Urine samples from participants were collected before and after the first, sixth, and twelfth interaction sessions of each bonding phase to obtain urinary OXT levels for each time point. These were selected to capture the early-phase response, mid-phase development, and end-phase outcomes while accounting for feasibility and resource constraints. Participants were asked to submit a urine sample of 10–30 ml in a sterile specimen cup before and after the selected sessions to establish a baseline urinary OXT level for measuring OXT level changes throughout a single session. All samples were collected during the afternoon (4 pm – 6 pm) to account for diurnal hormone cycles [51].

After each bonding phase, participants completed the online LAPS and RDQ for the respective bonding partner. They attended a scanning session in a 7T fMRI scanner at the Auburn University MRI Research Center, performing different tasks (note that the results for those fMRI studies will be reported in different publications).

### 2.4 Data analysis

#### 2.4.1 OXT data preparation.

To extract OXT levels from the collected urine, samples were pre-processed and subsequently analyzed with the Enzo Oxytocin ELISA Kit (https://www.enzolifesciences.com/).

Pre-Processing. Initially, 2 mL of urine was combined with 2 mL of 0.1% trifluoroacetic acid (TFA) in water, followed by centrifugation at 17,000 g for 10 minutes. The resultant supernatant was then applied to a Sep-Pak C18 Solid Phase Extraction (SPE) cartridge pre-conditioned with 1 mL of methanol (MeCN) and 10 ml of 0.1% TFA in water. After washing the cartridge with 10 ml of 0.1% TFA in water, the elution was performed using 3 mL of a solution consisting of 95% MeCN and 5% 0.1% TFA in water, allowing gravity to facilitate the process. The eluate was collected, and its volume was reduced under a nitrogen stream to approximately 300 µl before being lyophilized to dryness. These dried samples were stored at -80°C until further analysis.

Analysis with the Enzo Oxytocin ELISA Kit. The dried urine extracts were thawed at room temperature for the ELISA assay and reconstituted in 250 µl of assay buffer for duplicate assays. The reconstituted samples were vortex-mixed and centrifuged at 17,000 g for ten minutes. Assay standards were prepared by diluting a 10,000 pg/mL standard in assay buffer. In each well of the ELISA plate, 100 µL of the standards, sample supernatants, and quality controls were added. Subsequently, 50 µL each of blue conjugate and yellow antibody were introduced into the wells, followed by an incubation period of 20 hours at 4°C. Post-incubation, the wells were emptied and washed thrice with wash buffer. Then, 200 µl of substrate solution was added, and the plate was incubated at room temperature for one hour without shaking. Finally, 50 µL of stop solution was added to each well, and the optical density was measured immediately at 405 nm and 570 nm using a BioTek ELX 808 ultra microplate reader with processing using Gen 5 software (both now Agilent, Santa Clara, CA).

#### 2.4.2 Variables of analyses.

To address the presented working hypotheses, the analyses encompassed three dependent (social bonding indicated by urinary oxytocin level, self-reported level of attachment, and social companionship evaluation towards each bonding partner) and three independent (type and order of bonding partner, urine collection time point) variables.

Dependent Variables. The difference between the OXT level before and after an interaction session was used to represent sessional OXT level development, abbreviated as SOLD (Sessional Oxytocin Level Difference). The LAPS was evaluated by summing up all numerical ratings to a total score for each bonding partner respectively. The original three subscales of the LAPS were not separately examined due to the implemented item changes as described above. The RDQ was used to extract answers to one dichotomous closed-ended question of the belief whether robot dogs could live up to companion dogs (Q23) and two open-ended questions that capture the perceived advantages of each bonding partner compared to one another (Q25 and Q26).

Independent Variables. Partner constituted a within-subjects variable and indicated the agent (Dog, AIBO) used in the bonding phase. The order serves as a between-subjects factor to account for the potential effects of partner order (Dog 1^st^, AIBO 1^st^). Time poses a second within-subjects variable representing the stage (Begin, Middle, End) of each bonding phase in which urinary samples were collected.

Covariates. Covariate variables included the personality trait Openness to experience, the intake of birth control medication, and the last menstrual cycle phase. The menstrual cycle phase, derived from the information on the beginning of the last menstrual cycle and the intake of birth control medication, was aggregated in a single variable named hormonal phase because oral contraceptive pills contain estrogen and/or progesterone, leading to a steady hormonal balance if taken consistently and, therefore, make up another hormonal composition comparable to menstrual cycle phases, both potentially influencing OXT levels [51].

#### 2.4.3 Statistical analyses.

The data analysis was conducted with a significant threshold of a p-value of 0.05 (two-tailed) using IBM Statistical Package for the Social Sciences Statistics (SPSS, Version 23). Residuals were tested for normality using the Shapiro-Wilk and Kolmogorov-Smirnov tests, and visual examination was done via residual histograms and quantile-quantile plots. Residual heteroscedasticity was visually assessed by examining a scatterplot of predicted values against residuals. Cohen’s d was calculated as an effect size measure for significant t-test results (small effect: *d* = 0.20; medium effect: *d* = 0.50; large effect: *d* = 0.80).

Neurobiological Level. To test H-I, whether urinary OXT levels were higher during the bonding phase with dogs compared to AIBOs, a fixed-effects model analysis was performed, including SOLD as dependent and Time (Begin, Middle, End), Partner (Dog, AIBO), and Order (Dog 1^st^, AIBO 1^st^) as independent variables. Openness and hormonal state were included as covariates in the analysis. For the planned follow-up comparison of the bonding partners over time, paired samples t-tests were performed contrasting SOLD between interaction sessions with a dog and an AIBO for each Time (Begin, Middle, End).

Emotional Level. To test H-II, whether the self-reported attachment was higher to dogs compared to AIBOs, a fixed-effects model analysis was performed, including the LAPS sum score as dependent and Partner (Dog, AIBO) and Order (Dog 1^st^, AIBO 1^st^) as independent variables. Openness was included as a covariate. For the planned follow-up comparison of the attachment towards the bonding partners, a paired samples t-test was performed.

Evaluative level. To assess H-III and whether social companionship evaluations were more positive for dogs than AIBOs, the selected items of the RDQ were analyzed. Collected responses to the dichotomous closed-ended question were evaluated by comparing the frequency of each response option. A binary logistic regression analysis was conducted to investigate the influence of Openness on response choice, with Openness as the predictor variable and response as the outcome variable. For the evaluation of the qualitative data extracted from responses to the selected open-ended questions, an inductive, semantic thematic analysis was conducted by one main analyst following the six-step guide by Braun and Clarke [52] (Table 1). In this recursive analysis process, individual responses were coded and iteratively grouped into overarching themes portraying underlying arguments for and against both Dog and AIBO as social companions.

**Table 1:** Phases of Thematic Analysis by Braun and Clarke (2006).

Phase	Description
1. Data familiarization	Repeatedly reading data
2. Data coding	Systematically coding data
3. Searching Themes	Collating codes into themes
4. Reviewing Themes	Reviewing themes in relation to initial data and data codes, generating a thematic analysis map
5. Refining and naming themes	Refining and mapping themes, generating definitions and names for each theme
6. Reporting	Reporting on and visualizing findings

*Note*. Adapted from “Using thematic analysis in psychology” by V. Braun & V. Clarke, 2006, *Qualitative RESEARCH in Psychology, 3*(2), 77–101. Copyright 2006 by Edward Arnold (Publishers) Ltd.

## 3. Results

### 3.1 Neurobiological level: Social bonding indexed by urinary oxytocin

**Fig 4:**
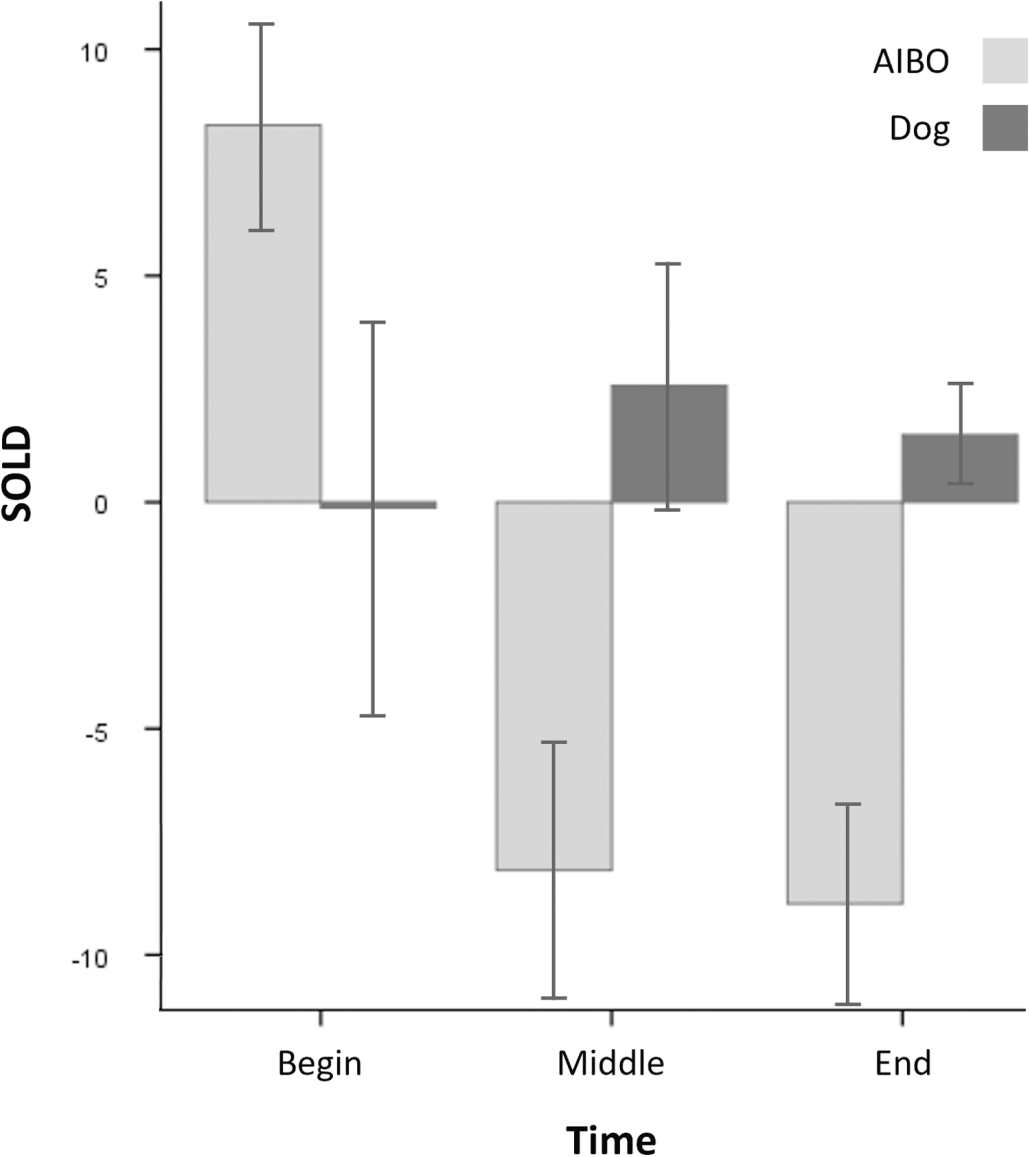
Average SOLD for Bonding Partners over Time. Average SOLD are shown for Partner (Dog, AIBO) over Time (Begin, Middle, End). Error bars represent the mean standard errors.

Testing for H-I, whether urinary OXT levels are higher during the bonding phase with dogs compared to AIBOs, the fixed-effects model analysis showed a significant main effect of Order (*F*(1, 47) = 8.32, *p *= .006) and significant interaction effects of Time x Partner (*F*(2, 37) = 7.09, *p *= .002) and Partner x Time x Openness (*F*(2, 37) = 7.13, p = .002). The planned follow-up comparisons of the Partner types using estimated means adjusted for Openness and hormonal state revealed non-significant differences at the Begin (*t*_*1*_(8) = 0.857, *p*_*1*_ = .416) and Middle (*t*_*2*_(8) = -1.295, *p*_*2*_ = .231) but a marginally significant difference at the End (*t*_*3*_(7) = -2.193, *p*_*3*_ = .064) of the bonding phase (Fig 4).

**Fig 5:**
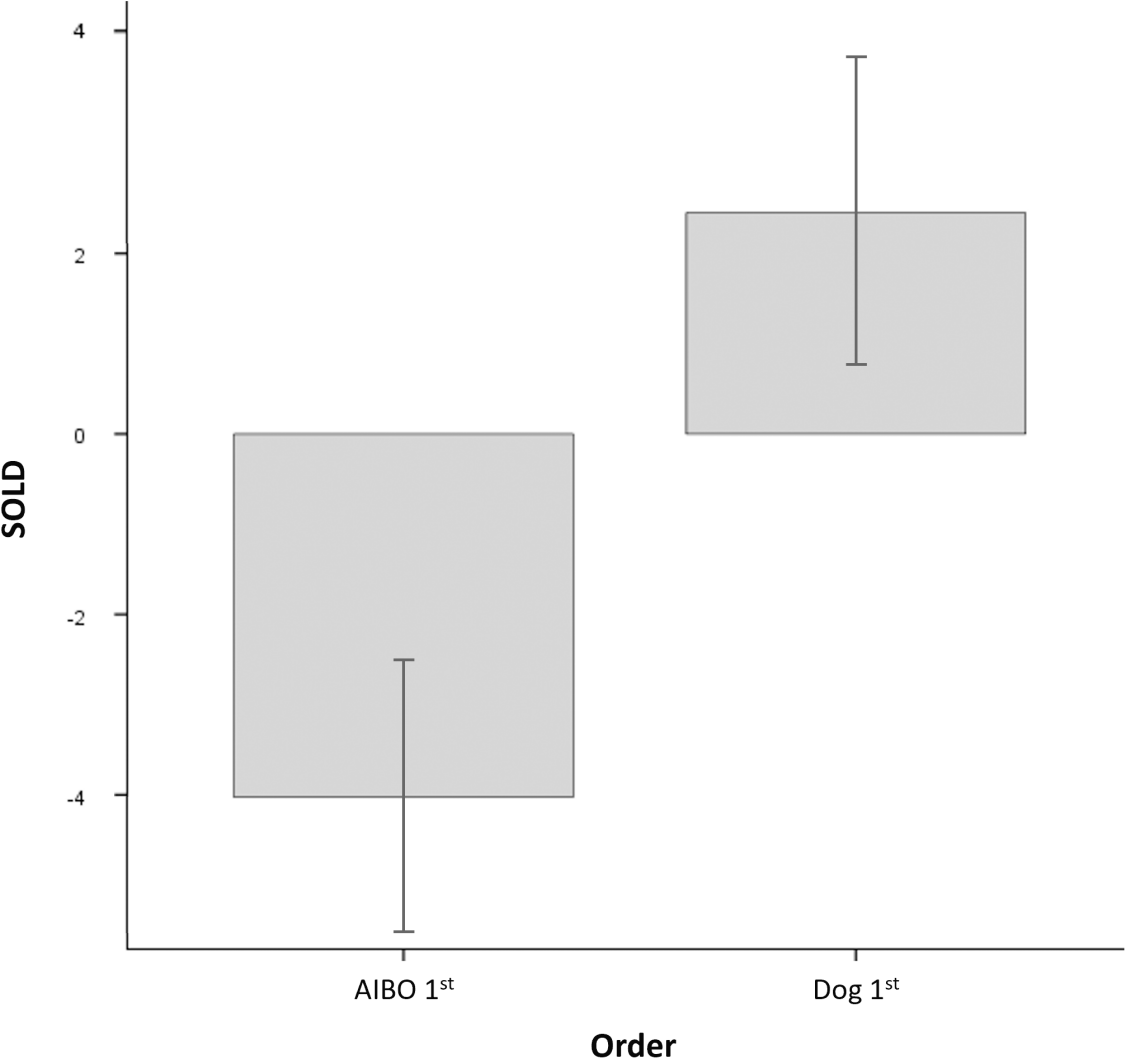
Average SOLD for Order of Bonding Partners. Average SOLD are shown for Order (Dog 1^st^, AIBO 1^st^). Error bars represent the mean standard errors.

To better understand the direction of the Order main effect, see Fig 5 suggesting an overall lower SOLD when the partnered AIBO was first encountered compared to when the partnered dog was first encountered.

To further explore the direction of the interaction effect of Partner x Time x Openness, a fixed-effects model analysis was additionally performed for each stage of Time (Begin, Middle, End) in the same manner as for the main analysis without Time as a factor. The interaction effect of Partner x Openness was not significant at the Begin (*F*(1, 16) = 0.25, *p *= .876) but reached significance at the Middle (*F*(1, 17) = 6.55, *p *= .020) and the End (*F*(1, 14) = 8.16, *p *= .012) of the bonding phase, indicating significant interactions of Partner and Openness over time.

To better understand the direction of this interaction effect, Spearman correlations between Openness and SOLD were post-hoc calculated and compared across partner types for the time stages Middle and End of the bonding phase with z-tests for paired samples. The comparisons showed that the correlations of Openness and SOLD did not significantly differ between Partner types during the Middle (*r*_*D*_ = -.35, *r*_*A*_ = .-.1, *z* = -.56, *p *= .286) nor End (*r*_*D*_ = .34, *r*_*A*_ = -.46, *z* = 1.564, *p *= .06) of bonding phase indicating no significant difference between the associations of Openness and SOLD depending on Partner Type at the Middle nor End.

### 3.2 Emotional level: Social bonding indexed by self-reported attachment (H-II)

**Fig 6:**
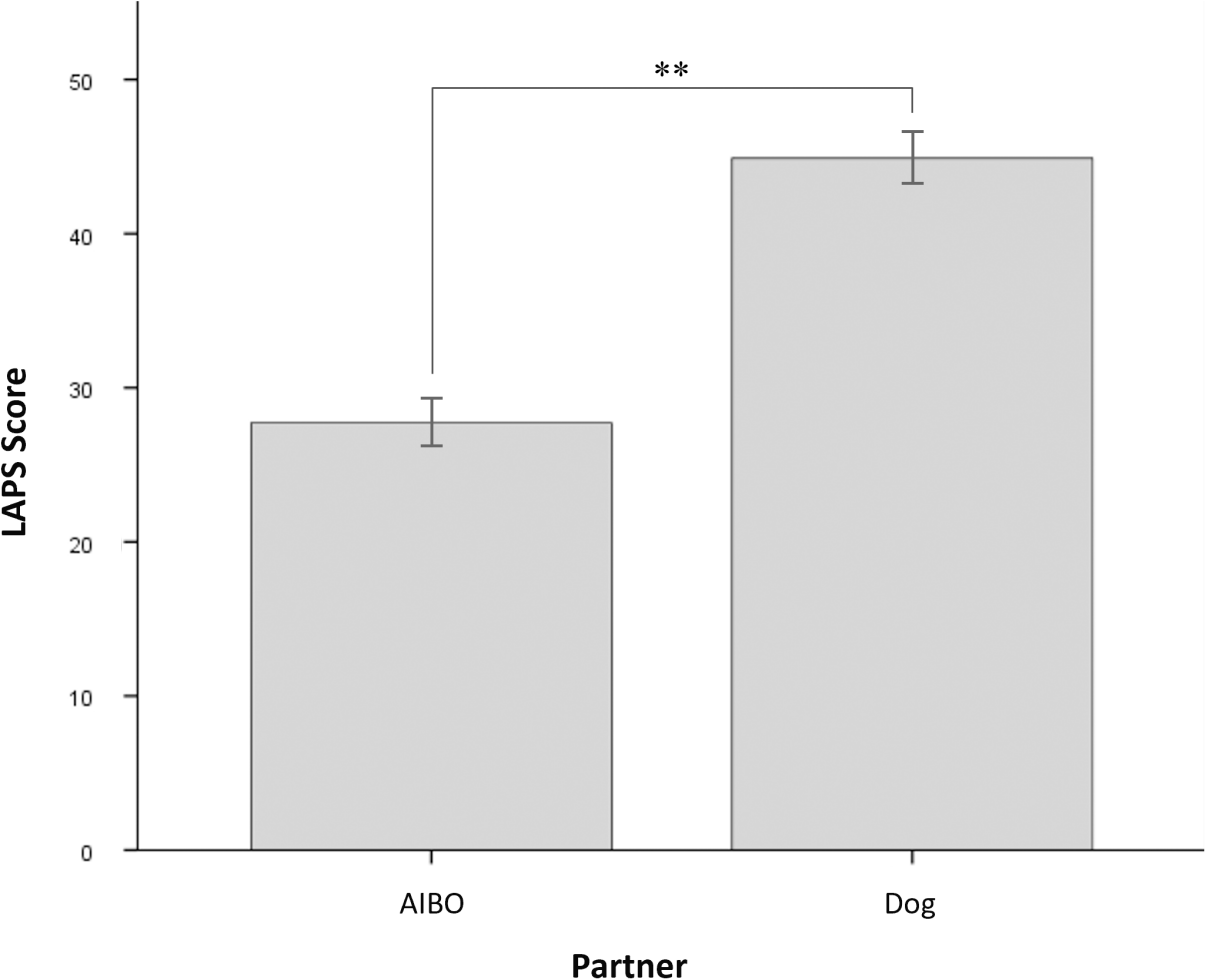
Average LAPS Sum Score for Bonding Partners. Average LAPS sum scores are shown for Partner (Dog, AIBO). Error bars represent the mean, standard errors. ** *p* < .01 (two-tailed).

Testing whether the self-reported attachment is higher in dogs compared to AIBOs, the fixed-effects model analysis did not show any significant main or interaction effects. However, the paired-samples t-test comparing the attachment towards the two bonding partners using estimated means adjusted for Openness revealed a significant difference between Dogs and AIBOs (*t*(17) = 3.67, *p* = .002; *d* = 1.02) (Fig 6).

### 3.3 Evaluative level: Social Bonding indexed by social companionship (H-III)

**Fig 7:**
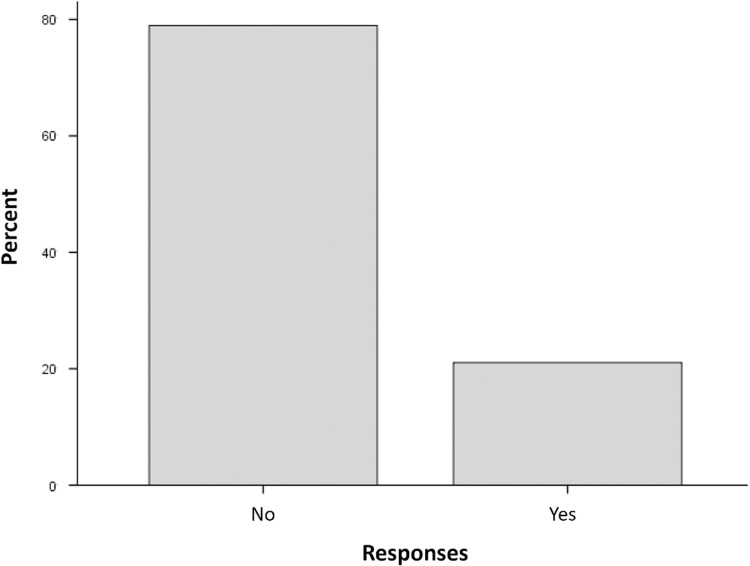
Frequency of Responses (RDQ-Q23). The percentage of the response options “Yes” and “No” to item Q23 of the RDQ (“Do you think a nice companion robot can be as good as a companion dog?”) are shown.

Testing whether social companionship evaluations are more positive for Dogs than AIBOs, the examination of the dichotomous closed-ended question (Q23) showed that 79% of the 38 collected responses indicated the belief that robot dogs would not be as good companions as dogs (*N* = 19) (Fig 7). From those participants who completed the RDQ more than once (*N* = 15), three shifted from the belief of equivalency to the belief of superiority of dogs over time, while two shifted inversely. The binary logistic regression testing the model of Openness as a predictor of the chosen response was not statistically significant (χ²(1, 37) = 0.21, *p* = .648), with the model explaining only 0.9% (Nagelkerke *R*^*2*^) of the variance in responses and 81.1% correctly classified cases.

**Fig 8:**
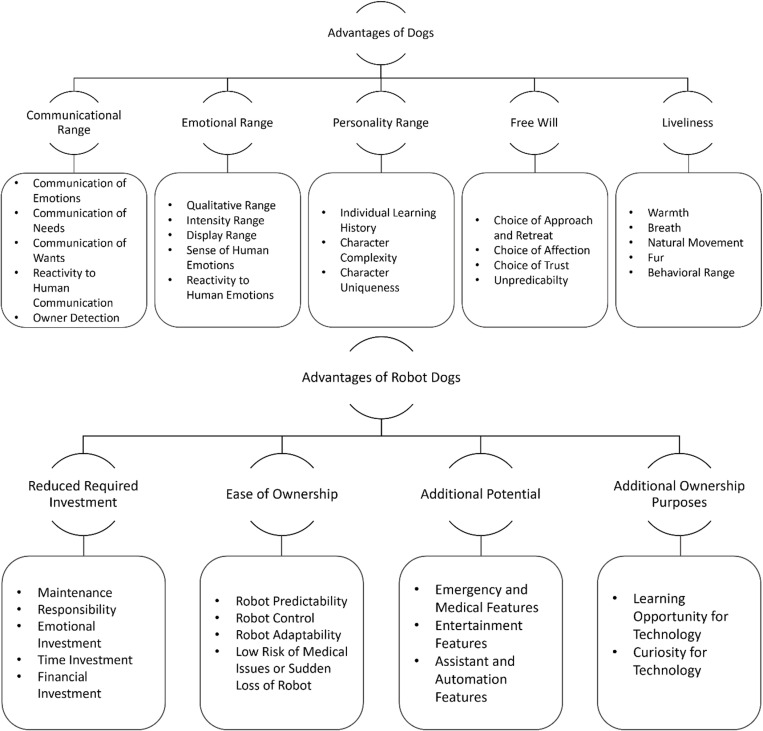
Thematic Analysis Map According to the Approach by Braun & Clarke (2006). The figure depicts the overarching advantages of dogs and AIBOs as social companions that were extracted from previously coded participant responses to the items Q25 (“List those features that make dogs better than robots, however skillful and cute the later are. “) and Q26 (“What kind of advantages would a well-functioning, intelligent dog-like robot have compared to a real dog?”) of the RDQ following the thematic analysis approach by Braun and Clarke [52]. The lowest hierarchy levels depict extracted response codes, the middle levels depict generated advantages, and the highest levels depict the social companion that is referred to.

Following the six-step guide by Braun and Clarke (52) for the evaluation of captured qualitative data, 23 codes for the advantages of dogs and 11 codes for the advantages of robot dogs were identified and grouped into five and four themes, respectively (Fig 8). Accordingly, advantages of dogs are their communicational, emotional and personality range, free will, and liveliness, while advantages of robot dogs encompass reduced required investment, ease of ownership, additional potential, and additional ownership purposes.

## 4. Discussion

In light of growing advancements in social robotic engineering, the question of successful social companionship provided by robots and the potential of interspecific social bonding arises. Our study explored social bonding between humans and an advanced companion robot called AIBO compared to dogs as well-established animated social companions on a neurobiological, emotional, and evaluative level. We hypothesized on the neurobiological, emotional, and evaluative level that both dogs and AIBOs would initiate social bonding but that dogs would enable a more profound connection. Our results supported our overall hypothesis. At the neurobiological level, serving as a neurobiological marker of social bonding, human urinary OXT levels significantly differed between interactions with dogs and AIBOs: OXT levels increased during affiliative interactions with dogs, whereas they decreased with AIBOs. At the emotional level, self-reported attachment, reflecting the emotional level of social bonding, was significantly higher for dogs than AIBOs. At the evaluative level, most participants believed that AIBOs could not match the social companionship provided by dogs. Additionally, the influence of Openness to experience on social bonding on all three levels remains inconclusive. Our findings indicate that social bonding with dogs works better over time than AIBO as a robotic social companion.

### 4.1 Social bonding in human-robot compared to human-animal interactions

Social Bonding Indexed by Urinary Oxytocin. Our prediction at the neurobiological level, assuming urinary OXT levels are higher during the bonding phase with dogs compared to robotic dogs, was partially confirmed. The average sessional OXT release did not significantly differ between dogs and AIBOs; however, a significant difference was observed over time. Sessional OXT release increased during the bonding phase when interacting with a dog and decreased when interacting with an AIBO. The difference in sessional OXT release between the types of bonding partners was marginally significant at the end of the bonding phase, while it remained insignificant at the beginning and middle, indicating an increasing divergence over time. Taken together, social bonding indexed by OXT seems to evolve progressively across all affiliative interactions with dogs, whereas interactions with AIBOs might initially induce the same process but lead to stagnation over time. This finding aligns with and extends previous research, which reported higher steady-state urinary OXT levels in individuals who lived with a companion robot than those living without any social companion [26]. While it was shown that social bonding with robotic social companions could be initiated, our study directly compared robots against dogs as well-established social companions and thus demonstrates the presently limited extent of social bonding with companion robots. The same study reported that eye contact with a temporarily owned companion robot does not increase urinary OXT levels. In contrast, another study showed that eye contact with an owned dog increased urinary OXT levels in its owner, further highlighting the current disparity between dogs and AIBOs as social companions [23]. Nonetheless, AIBOs and dogs seem to exhibit fewer differences as humans and their social companions start to become acquainted, which might indicate issues with sustaining social engagement in AIBOs or even in robotic social companions in general. Further research is warranted to unravel the reasons for the differences.

As found in previous research, Openness to experience appears to play a role in sessional OXT release over time, depending on the type of bonding partner. While Openness to experience and type of bonding partner did not significantly interact at the beginning of the bonding phase, significant interactions could be found in the middle and end of the bonding phase. However, the correlation between Openness to experience and sessional OXT release did not significantly differ between partner types for the middle and end of the bonding phase. Taken together, these findings are rather inconclusive and need further investigation with bigger sample sizes to explore the potential influence of Openness to experience on social bonding indexed by urinary OXT.

In the mixed effect model analysis, it was observed that sessional OXT release was higher across all time stages and partner types for participants who interacted first with a dog than for participants who interacted first with an AIBO. Interestingly, this main effect could not be found in the post-hoc independent samples t-test comparing sessional OXT between the interaction partner orders. Thus, further research is needed to unravel the role of the order of interaction partners in this paradigm.

Social Bonding Indexed by Self-reported Attachment. Our assumption at the emotional level that self-reported attachment is higher for dogs than robotic dogs was partially confirmed. The self-reported level of attachment after completion of the bonding phase was higher towards dogs than AIBOs. Interestingly, the fixed-effects model analysis did not reveal significant main or interaction effects of Openness to experience, type, or order of bonding partners. This apparent discrepancy between the t-test and the results of the fixed-effects model analysis may be grounded in the small sample size and, therefore, the limited power to detect significant effects when testing multiple factors and interactions simultaneously in the fixed-effects model analysis. Nonetheless, an overall higher attachment towards dogs aligns with the findings involving sessional OXT release, suggesting a more successful experience of social bonding with dogs than AIBOs.

Social Bonding Indexed by Social Companionship. Our assumption at the evaluative level was confirmed: dogs receive more positive evaluations of social companionship than robotic dogs. Most participants considered AIBOs inferior to dogs as social companions in direct comparison. Equivalent to the findings involving self-reported attachment, Openness to experience did not play a significant role in the social companionship evaluation. AIBOs appear primarily as dog simulations instead of unique entities with dog-like and dog-distinct features and abilities. Participants emphasized the genuineness of dogs’ emotions, behaviors, and attachment by contrast with AIBOs, whose output was perceived as a manufactured simulation of the former (“With robots, the human is mainly controlling the robot, which is not genuine.”). This fits in with previous research which found that most people do not believe in the equivalency of robots and dogs as social companions [42]. Even though companion robots are considered socially engaging, they are still perceived as technical artifacts and inferior compared to dogs that are favored for their emotions, personality, and attachment display [43,44].

Moreover, our findings are congruent with the overarching advantages of dogs found in the thematic analysis, encompassing communicational, emotional, and personality range, free will, and liveliness. While the illusion of dog-like liveliness, communication, emotions, and personality can be improved by bettering animation (e.g., fur, fluent movement, simulation of body heat and breath) and adding more complexity and variability to the latter three (e.g., extension of sensory systems, pre-programmed behavior, and response algorithms), a genuinely free will based on emotional and personal experiences independent of its designers’ predetermination cannot be given to an AI-powered robot yet. In addition, the appearance of AIBOs imitates the basic aesthetics of dogs (e.g., body composition and proportions) while maintaining a distinct artificial look (e.g., smooth surface, visible body part segmentation, neotenous design with large, expressive eyes and soft, rounded shapes), may have contributed to the perception of AIBOs as technical artifacts and the advance of dogs as social companions by limiting the illusion of animacy.

However, the chosen study design compared dogs and AIBOs as social companions in activities that were matched to the abilities of dogs. Hence, the full range of AIBOs’ capabilities, some of which exceed dogs’ qualities (e.g., access to the internet, the ability to take pictures, dancing, and playing music), might have been neglected, leading to a general impression of inferiority compared to dogs. Additionally, only one participant indicated that she had not owned a dog before. Thus, the selected sample might have been biased due to previous experiences with their dogs and an assumably positive attitude towards dogs in general. Therefore, it is recommended to explore further the potential of companion robots such as AIBOs in future research, focusing on their unique, strong suits as reflected by the found advantages of robot dogs encompassing reduced required investment, ease of ownership, additional potential, and additional ownership purposes and concentrating on populations that cannot cohabit with dogs as social companions (e.g., due to health constraints).

Social Bonding and Openness to Experience. Across all investigated levels of social bonding, the role of Openness to experience remains inconclusive. While some of our fixed effects model analyses revealed a significant effect of Openness to experience on sessional OXT release over time contingent upon the type of bonding partner, we did not observe a significant difference in the correlation between Openness to experience and sessional OXT release across different partner types at individual time points. The fixed-effects model analysis found no significant main or interaction effect of Openness to experience on self-reported attachment to bonding partners. Similarly, the binary logistic regression testing Openness as a predictor of the self-reported belief that whether robot dogs could live up to companion dogs was insignificant. The role of Openness to experience needs to be further investigated utilizing bigger sample sizes to unravel the potential influence of Openness to experience on social bonding.

Altogether, we found evidence confirming our overall hypothesis, stating that both bonding partners would initiate social bonding, but dogs would enable a more profound connection. Dogs were shown to be more effective bonding partners on all investigated levels —neurobiological, emotional, and evaluative levels. While data referring to the emotional and evaluative level was not collected during the bonding phases, a sessional OXT release informing the neurobiological level was collected at the beginning, middle, and end of the bonding phases. Therefore, the increasing difference in sessional OXT release over time between the types of bonding partners crucially supports our overall hypothesis, showing that a positive trend in sessional OXT release developed over time for dogs but not for AIBOs. This indicates that humans form stronger social bonds with dogs than AIBOs over time. This aligns with the higher self-reported attachment to dogs compared to AIBOs after completion of the bonding phases and the more positive evaluation of dogs as social companions contrasted to AIBOs.

### 4.2 Limitations and future research

A few limitations inherent to our study should be acknowledged and addressed in future studies. One notable limitation of this study was the relatively small size and homogeneity of the sample, which consisted of 19 female Psychology undergraduate students from a single university in the US. A convenience sample of Psychology students, a group predominantly composed of females, was recruited for this study, resulting in the inclusion of only female participants [53]. The exclusive investigation of females enabled gender to be a constant variable, reducing extraneous data variation. Additionally, the menstrual cycle phase was recorded and included as a covariate in the neurobiological analyses to account for its known influence on OXT through hormonal fluctuations. However, previous research has shown that women generally hold less favorable attitudes toward social robots than men [54]. Moreover, de Graaf and Allouch [55] found that men and women prioritize different pre-conditions for human friendship formation when treating zoomorphic robots as social companions. Therefore, the inclusion of only female participants represents an important limitation of our study that must be considered when interpreting our findings. Future research must examine how gender differences influence the development of social bonds with companion robots. Additionally, disparities in perception, attitudes, and acceptance of social robots were observed across national cultures, highlighting the importance of investigating culturally diverse samples in further studies [56–58]. Moreover, most participants had previous experience with dog ownership and, thus, presumably high familiarity and affinity towards companion dogs. The limited number of participants risks the reliability of present findings and restricts the complexity of statistical models, necessitating multiple analyses. Future research with more diverse samples could provide a more reliable and comprehensive understanding of the dynamics between humans and robotic companions. Furthermore, future research might want to focus on specifically targeted populations that could benefit the most from robotic social companionship. Thus, they may be the most motivated to engage with robotic social companions, such as individuals of advanced age who cannot care for a companion dog. Exploring the potential of robotic social companionship in these populations might better inform the social bonding process with social robots and lead to inventions and improvements in practical applications.

Another limitation of this study was the restricted availability of data, primarily due to inconsistent adherence to questionnaire completion instructions and the unavailability of additional data recorded by AIBO. Fluctuating levels of compliance with questionnaire instructions led to variability in the completeness of data entries, thereby increasing the risk of selection bias. Furthermore, AIBO’s internal data, including microphone and camera input, facial recognition data, and logging of AIBO’s learning history, which could potentially provide valuable insights, remained inaccessible for analysis. These factors collectively constrained the extent of data available for analysis and interpretation in the present study. Therefore, replication of the study is recommended, focusing on implementing measures to enhance participant compliance with questionnaire instructions. Additionally, efforts should be made to access and utilize internal data recorded by AIBO through collaboration with Sony Corporation.

Furthermore, it is important to note that the results reported in this study are specific to comparing AIBOs and dogs in terms of their ability to elicit social bonding in humans. As such, these findings should be interpreted within the framework of AIBOs’ underlying design principles rather than generalized to all companion robots with differing design concepts. However, while the most effective design for a companion robot has yet to be discovered, our qualitative analysis highlights shortcomings in AIBOs’ ability to foster social bonding in humans, which could serve as a starting point for future HRI research.

Finally, the bonding phases, each lasting four weeks with three interactions, offered a limited timeframe to establish and foster social bonds. In a previous study that revealed positive results of social bonding between humans and companion robots, a period of at least two months was chosen to form social bonds [26]. A similar strong relationship was observed for dog-owners [23], who presumably knew their dogs for months to years. Furthermore, their participants lived with a companion robot, providing greater contact availability. In contrast, our study occurred in a controlled laboratory environment that may have deviated from social bonding in real-life scenarios and focused on assessing the formation of bonding as opposed to measuring established bonds. To address these concerns in future research, the bonding phases could be extended, and the study could be transitioned to a more naturalistic setting, such as participants’ homes, where dogs and AIBOs could serve as temporary companions [59]. These adjustments can enhance ecological validity and provide a more accurate representation of bonding dynamics in real-world environments. While the discussed limitations should be kept in mind and require further attention in future research, this study has nonetheless contributed valuable new insights into comparing human social bonding with robotic and real dogs as social companions.

### 4.3 Summary and conclusion

Our study compared human social bonding with dogs and AIBOs, examining OXT levels, self-reported attachment, and evaluation of social companionship qualities. Our overall hypothesis that both bonding partners would initiate social bonding but that dogs would enable a more profound connection was confirmed. OXT release during interactions with dogs increased over time, whereas it decreased with AIBOs. Participants consistently reported stronger attachment to dogs and perceived them as superior social companions compared to AIBOs.

This is the first study to investigate social bonding between humans and robots serving as social companions in immediate comparison to dogs. We were able to show that dogs are presently more effective social companions in forming social bonds than robots on a neurobiological, emotional, and evaluative level. Therefore, this study contributes to the growing body of research in the field of HRI and helps to clarify the somewhat conflicting results reported by previous studies about the success of social bonding with companion robots with and without the contrast of a well-established, animated social companion.

In conclusion, our study provides valuable insights into human-robot bonding following the approach of ethorobotics, demonstrating that social robots designed to emulate dogs as models for social companionship currently fall short in immediate comparison to companion dogs. Social robots such as AIBOs might benefit from adopting distinct roles reflecting their technological capabilities to complement rather than imitate existing social companions. However, further research is needed to test this supposition and unravel key mechanisms driving social bonding with companion robots.
